# Thermal Activation of Thin Filaments in Striated Muscle

**DOI:** 10.3389/fphys.2020.00278

**Published:** 2020-04-16

**Authors:** Shuya Ishii, Kotaro Oyama, Seine A. Shintani, Fuyu Kobirumaki-Shimozawa, Shin’ichi Ishiwata, Norio Fukuda

**Affiliations:** ^1^Department of Cell Physiology, The Jikei University School of Medicine, Tokyo, Japan; ^2^Quantum Beam Science Research Directorate, National Institutes for Quantum and Radiological Science and Technology, Gunma, Japan; ^3^PRESTO, Japan Science and Technology Agency, Saitama, Japan; ^4^Department of Biomedical Sciences, College of Life and Health Sciences, Chubu University, Kasugai, Japan; ^5^Department of Physics, Faculty of Science and Engineering, Waseda University, Tokyo, Japan

**Keywords:** actomyosin, Ca^2+^ sensitivity, cardiac muscle, skeletal muscle, temperature, tropomyosin, troponin

## Abstract

In skeletal and cardiac muscles, contraction is triggered by an increase in the intracellular Ca^2+^ concentration. During Ca^2+^ transients, Ca^2+^-binding to troponin C shifts the “*on–off*” equilibrium of the thin filament state toward the “*on*” sate, promoting actomyosin interaction. Likewise, recent studies have revealed that the thin filament state is under the influence of temperature; *viz.*, an increase in temperature increases active force production. In this short review, we discuss the effects of temperature on the contractile performance of mammalian striated muscle at/around body temperature, focusing especially on the temperature-dependent shift of the “*on–off*” equilibrium of the thin filament state.

## Introduction

Under physiological conditions, striated muscle generates force and heat. Skeletal muscle plays a critical role in maintaining body temperature which increases during exercise. Human body temperature is maintained at ∼37 ± 1°C throughout the day (Refinetti, 2010; Geneva et al., 2019). In humans, body temperature rises to ∼39°C during exercise (Saltin et al., 1968) and exceeds ∼40°C during heat-related illnesses (e.g., heat stroke and malignant hyperthermia) (Glazer, 2005; Rosenberg et al., 2015). Physiologists have long perceived that a change in body temperature affects the mechanical properties of skeletal and cardiac muscles, such as active force generation and shortening velocity. However, the molecular mechanisms are yet to be fully understood, due, primarily, to the fact that sarcomere proteins have varying degrees of temperature sensitivity. Here, we briefly review the effects of temperature on the mechanical properties of skeletal and cardiac muscles in the range between ∼36 and ∼40°C, and discuss how striated muscle works efficiently at/around body temperature.

## Excitation–Contraction Coupling

Contraction of skeletal and cardiac muscles is initiated by depolarization of the sarcolemmal membrane. In skeletal muscle, sarcolemmal depolarization directly triggers the release of Ca^2+^ from the sarcoplasmic reticulum (SR) via ryanodine receptors; however, in cardiac muscle, it is the Ca^2+^ entry from the extracellular fluid through voltage-dependent L-type Ca^2+^ channels that triggers the Ca^2+^ release, a mechanism known as Ca^2+^-induced Ca^2+^ release (Bers, 2002; Endo, 2009). In both skeletal and cardiac muscles, an increase in the intracellular Ca^2+^concentration ([Ca^2+^]_*i*_) promotes Ca^2+^ binding to troponin C (TnC) on thin filaments (Fukuda et al., 2009; Kobirumaki-Shimozawa et al., 2014). Unlike in skeletal muscle, cardiac myofilaments are not fully activated under physiological conditions because [Ca^2+^]_*i*_ is maintained relatively low (∼10^–6^ M), even at the peak of systole (Bers, 2002). Because of this partial activation nature, cardiac myofilaments exhibit non-linear properties, such as length-dependent activation (Kobirumaki-Shimozawa et al., 2014) and spontaneous sarcomeric auto-oscillations (SPOC) (see Ishiwata et al., 2011; Kagemoto et al., 2018). In both skeletal and cardiac muscles, lowering [Ca^2+^]_*i*_ dissociates Ca^2+^ from TnC, resulting in dissociation of myosin from thin filaments, i.e., relaxation.

## Ca^2+^-Dependent Activation of Thin Filaments

Ca^2+^-activated muscle contraction is mediated by regulatory proteins, i.e., troponin (Tn) and tropomyosin (Tm), which form a complex on actin filaments. At rest, the Tn–Tm complex prevents/weakens actomyosin interaction. At this “*off*” state, the carboxyl-terminal domain of TnI strongly binds to actin, and Tm blocks myosin binding to actin and/or force production of bound myosin. When [Ca^2+^]_*i*_ is increased, Ca^2+^-bound TnC interacts with TnI, and the carboxyl-terminal domain of TnI is dissociated from actin. The Tn conformational changes result in displacement of Tm on actin, which subsequently induces myosin binding to actin and force generation (e.g., Haselgrove, 1973; Huxley, 1973; Lehman et al., 1994; Vibert et al., 1997; Xu et al., 1999; Fukuda et al., 2009; Risi et al., 2017; Matusovsky et al., 2019). It has been reported that during the shift of the thin filament state from “*off*” to “*on*,” strongly bound myosin cooperatively enhances binding of neighboring myosin molecules that have ATP and thereby potentially produce force (Greene and Eisenberg, 1980; Trybus and Taylor, 1980).

## Modulation of Myofibrillar Ca^2+^ Sensitivity

The “*on–off*” equilibrium of the thin filament state is most typically reflected as Ca^2+^ sensitivity of active force development in skinned fibers. The parameter pCa_50_ (= −log[Ca^2+^]) (required for half-maximal Ca^2+^-activated force) is widely used to express Ca^2+^ sensitivity; an increase in the pCa_50_ value indicates an increase in Ca^2+^ sensitivity and *vice versa*. Ca^2+^ sensitivity is influenced by various factors, such as the intracellular concentrations of Mg^2+^ (Fabiato and Fabiato, 1975; Best et al., 1977; Donaldson et al., 1978), MgATP (Fabiato and Fabiato, 1975; Best et al., 1977), MgADP (Fukuda et al., 1998, 2000) and inorganic phosphate (Kentish, 1986; Millar and Homsher, 1990; Fukuda et al., 1998, 2001), and ionic strength (Kentish, 1984; Fink et al., 1986) and pH (Fabiato and Fabiato, 1978; Orchard and Kentish, 1990; Fukuda and Ishiwata, 1999; Fukuda et al., 2001). Ca^2+^ sensitivity is likewise under the influence of phosphorylation/dephosphorylation of thick or thin filament proteins. Most importantly, protein kinase A, activated upon β-adrenergic stimulation in cardiac muscle, phosphorylates TnI, resulting in a decrease in Ca^2+^ sensitivity via weakening of the TnI–TnC interaction (see Solaro and Rarick, 1998 for details). Likewise, other translational modifications such as glycation (Papadaki et al., 2018) and acetylation (Gupta et al., 2008) may affect Ca^2+^ sensitivity.

## Effects of Temperature on the Mechanical Properties of Cardiac Muscle

**FIGURE 1:**
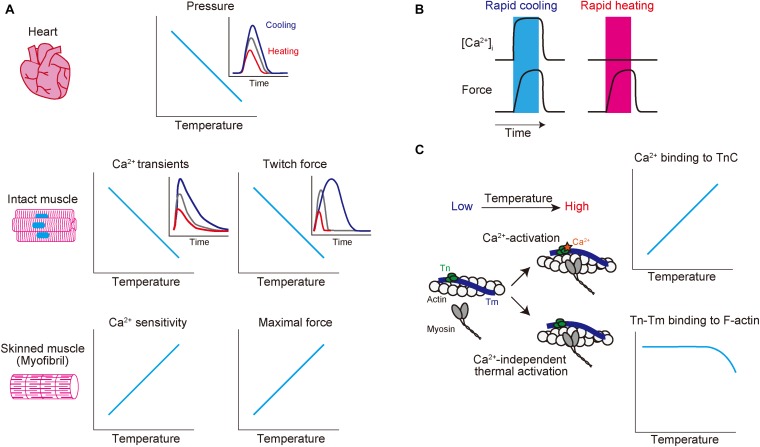
Schematic showing the effects of altered temperature on functional properties of mammalian striated muscle. **(A)** Top: relationship of temperature vs. endo-systolic pressure in mammalian hearts. Inset: time-course of ventricular pressure at different temperatures. Blue, gray, and red lines indicate hypothermic, physiological, and hyperthermic conditions, respectively. Middle: relationship of temperature vs. Ca^2+^ transients (left) and twitch force (right) in intact muscles. Blue, gray, and red lines indicate hypothermic, physiological, and hyperthermic conditions, respectively. Bottom: relationship of temperature vs. Ca^2+^ sensitivity (left) and maximal force (right) in skinned muscles. **(B)** Effects of rapid cooling (left; shown in blue bar) or rapid heating (right; shown in red bar) on [Ca^2+^]_*i*_ (top) and force (bottom) in intact cardiomyocytes. Rapid cooling increases both [Ca^2+^]_*i*_ and force, while rapid heating increases force with little or no influence on [Ca^2+^]_*i*_. **(C)** Effects of a change in temperature on thin filaments. Increasing temperature 1) enhances Ca^2+^ binding to TnC (see top graph) and 2) induces Ca^2+^-independent thermal activation of thin filaments via partial dissociation of the Tn–Tm complex from actin (see bottom graph), thereby coordinately acting to increase the fraction of the “*on*” state of thin filaments. See text for details.

A rapid decrease in solution temperature generates contraction in intact cardiac muscle [rapid cooling contracture (RCC): see Kurihara and Sakai, 1985; Bridge, 1986]. The mechanism of RCC can be explained as follows: upon lowering of the solution temperature, Ca^2+^ is released from the SR via ryanodine receptors (Protasi et al., 2004), causing contraction in a Ca^2+^-dependent manner. Chronic cooling also enhances contraction in intact cardiac muscle under varying experimental conditions (hypothermic inotropy) (Shattock and Bers, 1987; Puglisi et al., 1996; Mikane et al., 1999; Janssen et al., 2002; Hiranandani et al., 2006; Shutt and Howlett, 2008; Obata et al., 2018) (see Figure 1A and Table 1 for effects of alteration of temperature on striated muscle properties). For instance, Shattock and Bers (1987) reported that cooling from 37 to 25°C increases twitch force greater than approximately ∼fivefold in “intact” rabbit and rat ventricular muscle. However, cooling from 36 to 29°C *decreases* maximal Ca^2+^-activated force in “skinned” rabbit ventricular muscle, coupled presumably with depressed actomyosin ATPase activity, with no significant change in Ca^2+^ sensitivity (Harrison and Bers, 1989) (cooling to 22°C decreases both force production and Ca^2+^ sensitivity, see Table 1). We, therefore, consider that hypothermic inotropy is caused by the positive effect of cooling on [Ca^2+^]_*i*_ minus its negative effect on myofibrils: *viz.*, cooling increases the amplitude of the intracellular Ca^2+^ transients and prolongs the duration of the amplitude (i.e., longer time to peak [Ca^2+^]_*i*_ and slower [Ca^2+^]_*i*_ decline) (Puglisi et al., 1996; Janssen et al., 2002; Shutt and Howlett, 2008), hence, augmenting contractility in a Ca^2+^-dependent manner, by a magnitude greater than the decrease at the myofibrillar level.

**TABLE 1:** Effects of alteration of temperature on functional properties of mammalian striated muscles.

**Preparation**	**Parameter**	**Temperature change (°C)**	**Change in parameter value**	**Direction, change in parameter value**	**References**
Canine heart (isolated)	Systolic pressure (mmHg)	35.9→30.7 35.9→39.8	69.4→102.0* 69.4→44.8*	Increase Decrease	Mikane et al., 1999
Canine heart (isolated)	Systolic pressure (mmHg)	36.3→41	125.1→80.5***	Decrease	Saeki et al., 2000
Rat heart (isolated)	Systolic pressure (mmHg)	37→32 37→42	103.4→134.6* 103.4→76.0*	Increase Decrease	Obata et al., 2018
Guinea pig cardiac muscle	Force	36.5→17	–	Increase	Kurihara and Sakai, 1985
Rabbit cardiac muscle	Force	30→1	–	Increase	Bridge, 1986
Rabbit cardiac muscle	Twitch force	37→25	–	Increase	Shattock and Bers, 1987
Rat cardiac muscle	Twitch force	37→25	–	Increase	Shattock and Bers, 1987
Rat cardiac muscle	Twitch force (mN/mm^2^)	37.5→30	30→86	Increase	Janssen et al., 2002
Rat cardiac muscle	Twitch force (%, normalized at 37°C)	37→32 37→42	– 100→67.2	Increase Decrease	Hiranandani et al., 2006
Rabbit cardiac muscle	Twitch shortening (%)	35→25	7.6→13.1**	Increase	Puglisi et al., 1996
Ferret cardiac muscle	Twitch shortening (%)	35→25	2.9→4.9*	Increase	Puglisi et al., 1996
Cat cardiac muscle	Twitch shortening (%)	35→25	10.8→6.0*	Decrease	Puglisi et al., 1996
Guinea pig cardiac muscle	Twitch shortening (%)	37→22	2.6→8.3*	Increase	Shutt and Howlett, 2008
Rabbit cardiac muscle (skinned)	Maximal force (%, normalized at 22°C)	36→29 36→22	118.5→108* 118.5→100**	Decrease Decrease	Harrison and Bers, 1989
Rat skeletal fiber	Resting force (intact) Resting force (skinned)	30→40 30→40	– –	Increase Increase	Ranatunga, 1994
Rat cardiac muscle	Shortening	36→41	–	Increase	Oyama et al., 2012
C2C12 myotube	Shortening (%)	36.5→41.5	0→2.4*	Increase	Marino et al., 2017
Rabbit cardiac muscle	Ca^2+^ transient amplitude (nM)	35→25	248→454**	Increase	Puglisi et al., 1996
Rat cardiac muscle	Ca^2+^ transient amplitude (μM)	37.5→30	0.73→1.33	Increase	Janssen et al., 2002
Guinea pig cardiac muscle	Ca^2+^ transient amplitude (nM)	37→22	35→157*	Increase	Shutt and Howlett, 2008
Actin (RS) + Tm (HC) + Tn (HC) + HMM (RS)	Sliding velocity at pCa 5 Sliding velocity at pCa 9 Sliding velocity at pCa 9	∼20→∼60 ∼20→∼43 ∼43→∼60	– – –	Increase No change Increase	Brunet et al., 2012
Actin (RS) + Tm (HC) + Tn (BC) + HMM (RS)	Sliding velocity at pCa 5 (μm/s) Sliding velocity at pCa 9 (μm/s)	25→41.0 25→40.8	6.4→17.9 0→14.5	Increase Increase	Ishii et al., 2019
Actin (RS) + Tm (HC) + Tn (BC) + HMM (BC)	Sliding velocity at pCa 5 (μm/s) Sliding velocity at pCa 9 (μm/s)	24→40.0 24→39.9	1.19→8.89 0→3.37	Increase Increase	Ishii et al., 2019
Rabbit cardiac muscle (skinned)	pCa_50_ (active force)	36→29 36→22	5.473→5.494 (NS) 5.473→5.340**	No change Decrease	Harrison and Bers, 1989
Rabbit skeletal myofibril	pCa_50_ (ATPase)	30→37	7.05→7.52	Increase	Mühlrad and Hegyi, 1965
Rabbit skeletal myofibril	pCa_50_ (ATPase)	30→40	–	Increase	Murphy and Hasselbach, 1968
TnC (BC)	pCa_50_ (Ca^2+^ binding)	21→37	5.29→5.42*	Increase	Gillis et al., 2000
TnC (HC)	pCa_50_ (Ca^2+^ binding)	30→45	5.04→5.17	Increase	Veltri et al., 2017

Data obtained under various experimental conditions are summarized. Sliding velocity was obtained in the *in vitro* motility assay with reconstituted thin filaments (actin plus Tn–Tm). BC, bovine cardiac; HC, human cardiac; HMM, heavy meromyosin; RS, rabbit skeletal. *P < 0.05; **P < 0.01; ***P < 0.001. NS, not significant.

In contrast, an increase in temperature to ∼40–42°C has been reported to decrease end-systolic pressure in canine (Mikane et al., 1999; Saeki et al., 2000) and rat (Obata et al., 2018) hearts. The findings of these studies were confirmed by a study using rat ventricular trabeculae where twitch force was decreased by ∼30% accompanied by an increase in temperature from 37 to 42°C (Hiranandani et al., 2006). The mechanisms of hyperthermic negative inotropy are yet to be clarified; however, a decrease in the peak or duration time of Ca^2+^ transients is likely to underlie the inhibited active force production.

## Heating-Induced Contraction in Resting Muscle

Physiologists have realized for nearly a century that despite being under resting conditions, the warming of muscles increases active force, known as “heat contraction” or “heat rigor”. For instance, Vernon (1899) investigated heat contraction in cardiac and skeletal muscles that had been obtained from 18 species of cold-blooded animals. Likewise, Hill (1970) reported in frog sartorius muscle that resting tension is increased in a linear fashion with increasing temperature from 0 to 23°C and more steeply in the higher temperature range. Later, using intact and skinned rabbit skeletal muscle fibers, Ranatunga (1994) confirmed Hill’s finding that resting force is increased in a linear fashion in the low temperature range, i.e., <∼25°C and more sharply increased in the higher temperature range (30–40°C).

Recently, we demonstrated that rapid and repetitive heating via infrared laser irradiation (0.2 s at 2.5 Hz) induces transient and reversible shortening in isolated intact rat ventricular myocytes (Oyama et al., 2012). In this previous study, at the baseline temperature of 36°C, the magnitude of the rise in temperature to induce myocyte shortening was ∼5°C. It is important that this temperature-dependent contraction occurs in a Ca^2+^-independent manner, and instead, it is regulated at the sarcomere level. Indeed, intracellular Ca^2+^ imaging with fluo-4 revealed little or no increase in [Ca^2+^]_*i*_ upon infrared laser irradiation, and heating-induced contraction was blocked by the myosin II inhibitor blebbistatin. A similar phenomenon was observed in C2C12 myotubes (from mouse) when temperature was increased from 36.5 to 41.5°C using gold nanoshells in combination with near-infrared laser irradiation (Marino et al., 2017). These studies using differing preparations indicate that a rise in temperature from physiological ∼37 to ∼40°C directly activates sarcomeres in a Ca^2+^-independent fashion (Figures 1B,C).

## Thermal Activation of Thin Filaments

The characteristics of heating-induced contraction are consistent with the notion that Ca^2+^ sensitivity is increased with increasing temperature above 37°C (e.g., Ranatunga, 1994; Oyama et al., 2012). Mühlrad and Hegyi (1965) reported that increasing temperature in the range of 0–37°C reduces [Ca^2+^] for half-maximal and maximal ATPase activity in rabbit skeletal myofibrils. Warming to ∼40°C further reduces [Ca^2+^] for half-maximal ATPase activity in rabbit skeletal myofibrils (i.e., increased Ca^2+^ sensitivity) (see Murphy and Hasselbach, 1968), and interestingly, the Ca^2+^ sensitivity is lost at ∼50°C (Hartshorne et al., 1972).

By taking advantage of the *in vitro* motility assay, recent studies confirmed heating-induced activation of thin filaments by measuring the sliding velocity of reconstituted thin filaments. Brunet et al. (2012) analyzed sliding movements of thin filaments that had been reconstituted with human cTn and Tm at temperatures above ∼43°C under relaxing conditions in the absence of Ca^2+^ (+EGTA). We performed a rapid-heating experiment using infrared laser irradiation and found that thin filaments that had been reconstituted with bovine cTn and human Tm exhibited sliding movements at >∼35°C in the absence of Ca^2+^ (Ishii et al., 2019). Because the sliding velocity was ∼30% at 37°C compared to the maximum, this previous finding suggests that thin filaments are partially activated in diastole at physiological body temperature, enabling rapid and efficient myocardial dynamics in systole (see Ishii et al., 2019 for details).

The molecular mechanisms of thermal activation of thin filaments are yet to be fully understood. One possible mechanism is “partial dissociation” of Tn–Tm from F-actin upon increasing temperature (as discussed in Oyama et al., 2012; Ishii et al., 2019); *viz.*, Tanaka and Oosawa (1971) demonstrated that Tm dissociates from F-actin at >∼40°C. Later experiments by Ishiwata (1978) on reconstituted thin filaments (F-actin plus Tn–Tm) showed that Tn–Tm starts to partially dissociate from F-actin at ∼41°C, with dissociation temperatures of 48.8 and 47.0°C in the absence and presence of Ca^2+^, respectively.

While in older studies the structural changes in thin filaments were unable to be detected, newer studies suggest that heating-induced Ca^2+^-independent contraction may result not only from partial dissociation of Tm or Tn–Tm from F-actin but also from structural changes in Tn, Tm, or both. Consistent with this view, Kremneva et al. (2003) reported that thermal unfolding occurs in Tm in reconstituted thin filaments comprised of F-actin and Tn–Tm. They found that a low-temperature transition reflecting the denaturation of the C-terminus of Tm started to occur at ∼40°C in the presence of 1 mM EGTA (hence under the relaxing condition). Likewise, it has previously been reported that instability of the coiled-coil structure of Tm is essential for optimal interaction with actin (Singh and Hitchcock-DeGregori, 2009). It is therefore likely that the unfolding of Tm may promote the shift of the thin filament state from the “*off*” state to the “*on*” state and thereby gives rise to, at least in part, heating-induced contraction.

It is likewise known that the Ca^2+^-binding affinity of TnC is increased with temperature. For instance, Gillis et al. (2000) reported that the Ca^2+^-binding affinity of bovine cardiac TnC is increased with temperature within the range between 7 and 37°C. The affinity of human cardiac TnC for Ca^2+^ also increases with temperature within the range between 21 and 45°C (Veltri et al., 2017). It should be noted that the temperature sensitivity of TnC for Ca^2+^ is isoform dependent. For instance, Harrison and Bers (1990) reported that the cooling-induced decrease in Ca^2+^ sensitivity is attenuated after reconstitution with skeletal TnC in skinned rat ventricular muscle.

## Possible Use of Local Heating for the Treatment of Dilated Cardiomyopathy

Accumulating evidence shows that mutations in sarcomere proteins, including Tn subunits (TnT, TnI, and TnC) and Tm, modulate Ca^2+^ sensitivity and thereby promote the pathogenesis of DCM or HCM (Ohtsuki and Morimoto, 2008). A general consensus has been achieved in that myofibril Ca^2+^ sensitivity is decreased by DCM mutations and increased by HCM mutations (Ohtsuki and Morimoto, 2008; Kobirumaki-Shimozawa et al., 2014). Because an increase in temperature enables sarcomeric contraction in a Ca^2+^-independent manner (Oyama et al., 2012), local heating, such as via infrared laser irradiation, may have a potential to augment contractility in patients with DCM without causing the intracellular Ca^2+^overload that can cause fatal arrhythmias. In order to avoid hyperthermal negative inotropy, local heating targeting myofibrils, but not global heating, is essential to augment contractility of myocardium in the heart. For instance, Marino et al. (2017) demonstrated gold nanoshell-mediated remote activation of myotubes via near-infrared laser irradiation, which does not cause a change in [Ca^2+^]_*i*_. Likewise, heating of nanoparticles by the magnetic field may be useful to increase temperature of the myocardium in various layers from the epicardium to the endocardium of the heart *in vivo* (as demonstrated by Chen et al., 2015 for deep brain stimulation).

It is worthwhile noting that in previous studies discussed, thus far, different mammals were used that have different body temperatures (cf. Table 1), thus, future studies using human samples need to be conducted under various experimental conditions to systematically investigate how alteration of temperature affects the function of the heart in humans.

## Conclusion

In striated muscle, sarcolemmal depolarization causes an increase in [Ca^2+^]_*i*_. The Ca^2+^-dependent structural changes of thin filaments allow for myosin binding to actin and thereby facilitate active force production. Cooling increases the contractility of striated muscle via Ca^2+^-dependent activation: first, a rapid decrease in temperature triggers a release of Ca^2+^ from the SR, and second, long-term cooling increases the amplitude as well as the period of intracellular Ca^2+^ transients. Contrary to these cooling effects, heating increases myofibrillar active force (and ATPase activity) and Ca^2+^ sensitivity; the latter is coupled with an increase in the affinity of TnC for Ca^2+^. Moreover, heating induces structural changes of thin filaments (i.e., partial dissociation of the Tn–Tm complex from F-actin), thereby shifting the “*on–off*” equilibrium of the thin filament state toward the “*on*” state at a given [Ca^2+^]_*i*_ (Ca^2+^-independent activation). The characteristics of heating-induced, Ca^2+^-independent activation may be useful to augment the heart’s contractility in patients with DCM in future clinical settings.

## Author Contributions

SIs, KO, and NF wrote the first version of the manuscript. SS, FK-S, and S’Is contributed comments and suggestions. All authors approved the final version of the manuscript.

## Conflict of Interest

The authors declare that the research was conducted in the absence of any commercial or financial relationships that could be construed as a potential conflict of interest.

## Abbreviations

[Ca^2+^]_*i*_: intracellular Ca^2+^ concentration
RCC: rapid cooling contracture
SPOC: spontaneous sarcomeric auto-oscillations
SR: sarcoplasmic reticulum
Tm: tropomyosin
Tn: troponin.
